# Interplay of Spin Nernst Effect and Entanglement Negativity in Layered Ferrimagnets: A Study via Exact Diagonalization

**DOI:** 10.3390/e26121060

**Published:** 2024-12-06

**Authors:** Leonardo S. Lima

**Affiliations:** Department of Physics, Federal Technological Education Center of Minas Gerais, Belo Horizonte 30510-000, MG, Brazil; lslima@cefetmg.br

**Keywords:** entanglement negativity, spin Nernst coefficient, exact diagonalization

## Abstract

In this paper, we analyzed the influence of the spin Nernst effect on quantum correlation in a layered ferrimagnetic model. In the study of three-dimensional ferrimagnets, the focus is on materials with a specific arrangement of spins, where the neighboring spins are parallel and the others are antiparallel. The anisotropic nature of these materials means that the interactions between spins depend on their relative orientations in different directions. We analyzed the effect of magnon bands induced by the coupling parameters on entanglement negativity. The influence of the coupling parameters of the topologic phase transition on quantum entanglement is investigated as well. Numerical simulations using the Lanczos algorithm and exact diagonalization for different lattice sizes are compared with the results of spin wave theory.

## 1. Introduction

Quantum correlation in low-dimensional magnets is an important topic in recent years that connects quantum information theory and condensed matter physics [1,2]. In the context of quantum spin systems, quantum correlation refers to the entanglement and non-classical correlations that exist between quantum particles. Ferrimagnets are intermediate states between ferromagnets and antiferromagnets. They hold significant relevance in condensed matter physics due to their unique magnetic properties, which emerge from the interplay of opposing magnetic moments on the different sublattices within a material. This inherent imbalance in magnetic moments offers intriguing opportunities in various modern applications, especially in spintronics, topological phenomena, and near-term quantum technologies, including noisy intermediate-scale quantum (NISQ) devices [1,3,4]. In three-dimensional ferrimagnets or layered ferrimagnets [5,6], the interplay between the topological phase transitions [7,8,9] and quantum correlations [2,10] gives rise to a range of intriguing phenomena, where entanglement plays a crucial role in determining the ground-state properties and various other exotic spin phenomena [11,12,13,14,15,16,17,18,19,20,21,22,23,24] observed in ferrimagnetic systems. On the other hand, the spin Nernst effect refers to the generation of an electric field perpendicular to both an applied temperature gradient and external field [25,26,27]. It is an important phenomenon that occurs in materials with broken time-reversal symmetry, being a variant of the standard Nernst effect, where the generated electric field is proportional to the spin current rather than the charge current. In the framework of layered ferrimagnets [5,6], it may emerge in the system due to the interplay between temperature gradients, magnetic fields, and spin transport. In general, when a temperature gradient is applied through a ferrimagnet, it may induce a spin current perpendicular to the temperature gradient, resulting in a transverse voltage known as spin Nernst voltage. This effect is directly related to the magnon Hall effect and can provide insights into the spin dynamics of ferrimagnetic materials. To study quantum correlations and the spin Nernst effect in a layered ferrimagnet, several theoretical models and computational techniques are employed, such as spin wave theory [28], density matrix renormalization group [29,30], quantum Monte Carlo [31,32], and exact diagonalization [33,34,35]. The predictions regarding quantum entanglement and the spin Nernst effect depend on the specific model, Hamiltonian, and the parameters used in the study, so the goal is to explore these phenomena experimentally and theoretically to gain a deeper understanding of the behavior of quantum spins in magnetic materials. In the layered ferrimagnet, the influence of magnon bands on quantum correlation is an interesting aspect to explore. The Heisenberg model describes the system with anisotropic and isotropic interactions, which is a widely studied model for understanding the behavior of layered ferrimagnets. In general, quantum correlations and entanglement in the Heisenberg model can be quantified using different quantifiers, such as von Neumann entanglement entropy or an entanglement spectrum. Indeed, von Neumann entropy provides information about the degree of entanglement between different regions of the spin system. The entanglement spectrum typically refers to the structure of the eigenvalues of the reduced density matrix on a bipartite system.

The presence of anisotropic interactions introduces a characteristic anisotropy in the magnon dispersion, which affects the entanglement dynamics, where the magnon bands determine the spectrum of the magnons, in turn affecting the entanglement dynamics. The von Neumann entropy and spectrum may exhibit different behaviors depending on the specific features of the magnon bands, such as their bandwidth, dispersion relation, and anisotropy. For example, the presence of gapless magnon modes can lead to long-range entanglement and power-law scaling regarding the von Neumann entropy. On the other hand, the presence of a magnon gap can suppress entanglement and lead to short-range correlations. The study of magnon bands and their influence on entanglement in the ferromagnetic and antiferromagnetic Heisenberg models often involves theoretical approaches such as spin wave theory, bosonization techniques, or numerical methods like exact diagonalization, which provide insights into the entanglement properties and their connection to the magnon spectrum of the system. Thus, an understanding of the interplay of magnon bands and entanglement is crucial for unraveling the quantum nature of ferrimagnetic systems, helping to characterize the ground-state properties and explore the emergence of novel phenomena in these systems [36,37,38,39,40,41,42,43,44,45,46].

The goal of this paper is to analyze the influence of variations in the spin Nernst coefficient with coupling parameters on entanglement negativity. The paper is organized as follows: in Section 2, we discuss the three-dimensional layered ferrimagnet and its properties. In Section 3, we present our analytical and numerical results, where we discuss the variations in the spin Nernst conductivity and the behavior of the entanglement negativity as a function of *T*. Finally, in Section 4, we present our conclusions.

## 2. Model

*General Hamiltonian for the layered ferrimagnet*: For a layered ferrimagnet system with two types of ferromagnetic sublayer *a* and *d* sublattices stacked periodically, we consider the Hamiltonian with intralayer ferromagnetic exchange interactions for each sublattice; interlayer antiferromagnetic coupling between adjacent sublayers; magnetic anisotropy for each sublattice; external magnetic field applied to the system; and dipole–dipole interaction as a perturbation to account for long-range dipolar effects. The model is composed by

(1) $$\begin{array}{c} H_{intra}=-\sum_{\langle l,i,l^{\prime },i\rangle \in a}J_{a}S_{a,i}\cdot S_{a,j}-\sum_{\langle l,i,l^{\prime },i\rangle \in d}J_{d}S_{d,i}\cdot S_{d,j}. \end{array}$$

Antiferromagnetic interlayer exchange coupling between neighboring layers:

(2) $$\begin{array}{c} H_{inter}=-\sum_{\langle l,i,l^{\prime },i\rangle }J_{0}S_{l,i}\cdot S_{l^{\prime },i}. \end{array}$$

Magnetic anisotropy for each sublattice:

(3) $$\begin{array}{c} H_{anisotropy}=-\sum_{(l,i)\in a}K_{a}{\left(S_{a,i}\cdot \hat{k}\right)}^{2}-\sum_{(d,i)\in d}K_{d}{\left(S_{d,i}\cdot \hat{k}\right)}^{2}. \end{array}$$

External magnetic field:

(4) $$\begin{array}{c} H_{field}=-\sum_{(l,i)\in a}B\cdot S_{a,i}-\sum_{(d,i)\in d}B\cdot S_{d,i}. \end{array}$$

Dipole–dipole interaction (perturbative term):

(5) $$\begin{array}{c} H_{dipole}=k\sum_{\left(l,i\right)\neq \left(l^{\prime },j\right)}\frac{S_{l,i}\cdot S_{l^{\prime },j}-3\left(S_{l,i}\cdot {\hat{r}}_{ij}^{ll^{\prime }}\right)\left(S_{l^{\prime },j}\cdot {\hat{r}}_{ij}^{ll^{\prime }}\right)}{|r_{ij}^{ll^{\prime }}{|}^{3}}, \end{array}$$

where $k=\mu_{0}{\left(g\mu_{B}\right)}^{2}/2$, $\mu_{0}$ is the vacuum permeability constant, *g* is the Landé factor, and $\mu_{B}$ is the Bohr magneton. $\hat{r}$ is the unit vector between spins *i* and *j*, and $|r_{ij}|$ is the distance between them. $B=\left(B_{x},B_{y},B_{z}\right)$ is the external magnetic field vector. Thus, the total Hamiltonian for the system is

(6) $$\begin{array}{c} H=H_{intra}+H_{inter}+H_{anisotropy}+H_{field}+H_{dipole}. \end{array}$$

The model captures the interactions within and between the two sublattices as well as the influence of anisotropy, external fields, and long-range dipolar effects.

## 3. Results

Entanglement is a quantum mechanical property that Schrödinger singled out as “the characteristic trait of quantum mechanics” and that has often been analyzed in connection with Bell’s inequality [47,48,49,50,51,52,53,54]. In general, a pure pair of quantum systems are called entangled whether or not they are unfactorable. It is well known that quantum information theory can be used together with condensed matter physics in characterizing quantum phase transitions (QPTs) that are characterized by the ground-state energy of quantum many-particle systems. The quantifying of quantum correlations in these many-body systems enhances the condensed matter physics and quantum information theory, being a measure of quantum correlation or entanglement in a system provided by the entanglement negativity [52,55].

### 3.1. Negativity

Negativity is defined as the linear and partial transpose whose trace norm is a convex and monotone function but not additive. Moreover, it presents a large deficiency, i.e., a failure in satisfying the discriminant property, such that the entanglement exists E(ρ)=0 if and only if ρ is separable [56]. The entanglement negativity [2,56,57] is provided for a mixed state $\rho_{GE}$ by

(7) $$\begin{array}{c} N\left(\rho \right)=\frac{∥\rho_{A}^{T}{∥}_{1}-1}{2}, \end{array}$$

where $\rho_{A}^{T}$ is the partial transpose of $\rho_{GE}$ with respect to the subsystem *A* and ${∥\cdots ∥}_{1}$ is the trace norm. The logarithmic negativity [58]

(8) $$E_{N}\left(\rho \right)=\log_{2}{∥\rho_{A}^{T}∥}_{1}$$

is often used as a measure of thermal entanglement for disjoint intervals. Consequently, the negativity has been proven to be useful to detect topological order [59,60], where one obtains $\rho_{A}=\rho_{GE}$, and we consider a bipartite lattice with N spins and in the following set N→∞ in the partition, with the aim of the spin wave approach being valid to obtain the entanglement negativity as

(9) $$\begin{array}{c} \;E_{N}=\frac{1}{k_{B}T}\sum_{\nu }\sum_{k}\left[\Omega_{\nu }{\left(k\right)}^{p}\left(\Omega_{\nu }\left(k\right)\right)+\log_{2}\left(1+e^{-\Omega_{\nu }\left(k\right)/k_{B}T}\right)\right], \end{array}$$

where the dispersion relation of magnons $\Omega_{\nu }\left(k\right)$ is provided in Appendix A and $p\left(x\right)={\left(e^{x/k_{B}T}-1\right)}^{-1}$ is the Bose–Einstein distribution.

### 3.2. Lanczos Algorithm and Exact Diagonalization

We obtain the entanglement negativity as a function of temperature for a finite lattice with L=256 sites using the Lanczos algorithm and exact diagonalization [33,34,35]. We perform a Python implementation using the Lanczos algorithm for approximate diagonalization combined with the entanglement negativity calculation as a function of temperature for a 2D lattice with 256 sites. After constructing the Hamiltonian matrix for the system, we use the Lanczos algorithm to compute the low-lying eigenstates and eigenvalues. By calculating the reduced density matrix by tracing out part of the system (half the lattice), we compute the entanglement negativity from the partial transpose of the reduced density matrix. The number of Lanczos iterations (set to 100 here) determines how many eigenvalues and eigenvectors are approximated, which depends on how many low-energy states are important for the thermal ensemble. The Lanczos method is efficient for large systems and helps in approximating the eigenstates without requiring full diagonalization. We obtain an increase in correlations $E_{N}$ at vanishing of *T*. This behavior is in accordance with the results obtained using basic linear algebra routines, where optimizations depending on the lattice size have provided large increases at ranges close to T∼0 as well. However, these results are different from results obtained by spin wave theory, where $E_{N}\to 0$ at T→0, since there is an inaccessible regime for mean field. Moreover, the results obtained by spin wave theory for higher temperature are only qualitative due to mean-field approach used.

**Figure 1:**
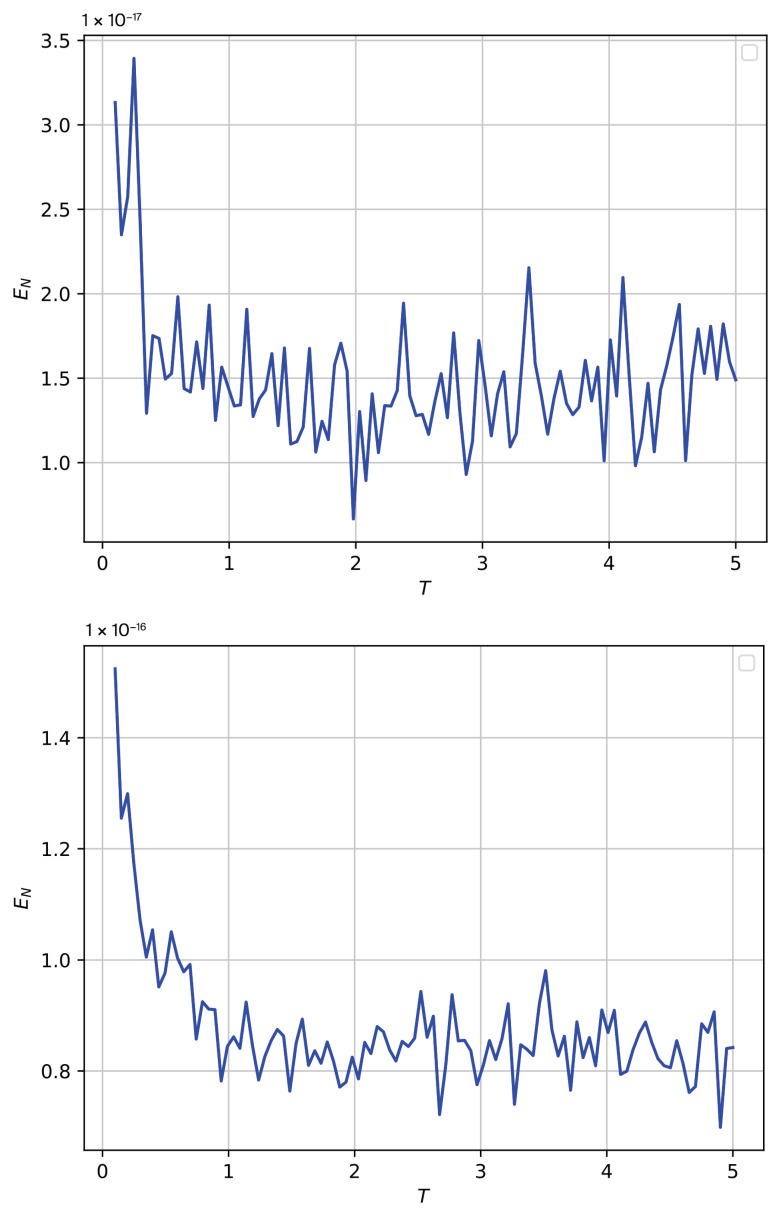
$E_{N}$ as a function of *T* using Lanczos algorithm and exact diagonalization. We consider two different finite size lattices, L=256 sites (above graphic) and L=1024 sites (below graphic). For all cases, we obtain a very small value for $E_{N}$ for all values of *T*, with $E_{N}$ tending to diverge at T=0 limit.

In Figure 1, regarding $E_{N}$ as a function of *T* using Lanczos algorithm and exact diagonalization, we consider two different finite size lattices, L=256 sites above graphic and L=1024 below graphic. For all cases, we obtain a very small value for $E_{N}$ for all values of *T*, with $E_{N}$ tending to diverge at T=0 limit. The results obtained are different from the results obtained using the spin wave approach, where $E_{N}\to 0$ at T=0. However, we hold that the results using the spin wave approach are valid in the continuum limit (N→∞), where we consider a partition with N spins for which the local negativity is calculated, and, in following, we set N→∞ in the partition with the aim of continuum theory being valid. The results obtained using exact diagonalization are valid for a finite lattice considering a bipartite lattice of finite size *L*. Moreover, the results from exact diagonalization access an inaccessible regime for mean field. The results from SWT are accurate at range of low *T*, the behavior being higher for temperatures that are only qualitative.

### 3.3. Analysis by SWT Approach

**Figure 2:**
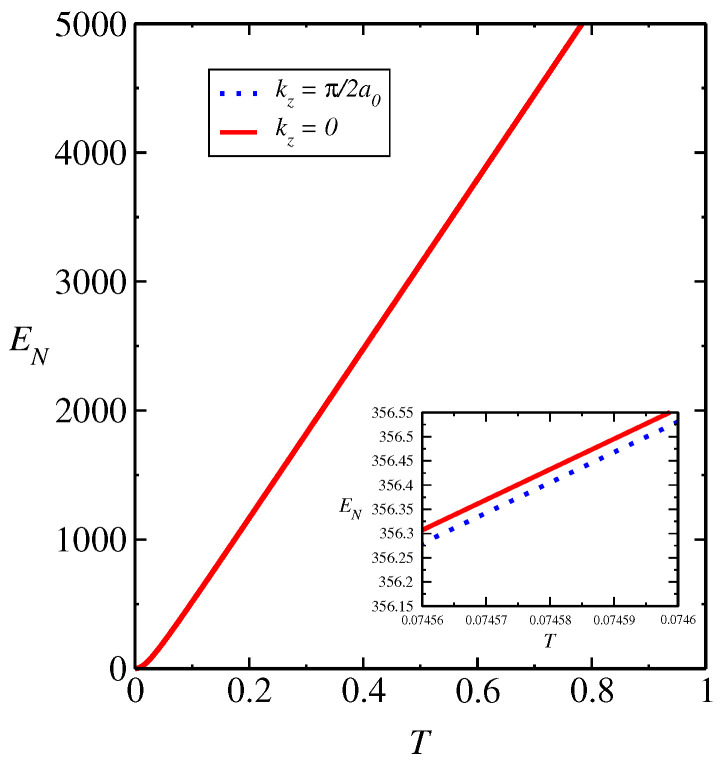
$E_{N}$ as a function of *T* by SWT approach for $k_{z}=\pi /2a_{0}$, which corresponds to the Brillouin zone edge in *z* direction (solid red line) and $k_{z}=0$ (dashed blue line). We obtain $\Omega^{\left\|(⊥\right)}=0.43\Omega_{ex}^{a}$. The value of $\Omega_{ex}^{a}=4.0$ corresponds to the value $J_{0}=0.8$ meV. We obtain a small difference in $E_{N}$ for the bands $\Omega_{\alpha }\left(k\right)$ and $\Omega_{\beta }\left(k\right)$.

In Appendix A, we describe the steps of diagonalization of the layered ferrimagnetic model with single-ion anisotropy *K* and external field using spin wave theory (SWT). In Figure 2, we obtain the entanglement negativity $E_{N}$ as a function of *T* using the SWT approach for $k_{z}=0$ and $k_{z}=\pi /2a_{0}$ that corresponds to the Brillouin zone edge in the *z* direction. We have $\Omega^{\left\|(⊥\right)}=0.43\Omega_{ex}^{a}$. As we obtained a very small difference in the spin wave spectra with (without) dipole–dipole interaction, as shown in Ref. [5], we must obtain a very small difference regarding entanglement negativity as well. Moreover, we obtain a small difference regarding behavior of $E_{N}$ on gap closing loop in $k_{z}=\pi /2a_{0}$ and $k_{z}=0$, where $E_{N}$ tends to zero at T→0 limit. In addition, we obtain that the small change in the curves of the entanglement negativity $E_{N}$ as a function of *T*, for different values $k_{z}=0$ and $k_{z}=\pi /2a_{0}$, in the gap closing loop and the system suffers a topological phase transition. A difference in negativity at the BZ edges indicates a qualitative change in the entanglement structure across momentum space, reflecting the topology of the phase. Furthermore, when a system undergoes a topological transition, the negativity may show discontinuities or non-analytic behavior at specific momenta (e.g., BZ edges) and the difference in negativity between the BZ edges may shift, signaling a change in the topological invariant of the system. Thus, the negativity difference at the edges of the BZ can act as a signature for topological phases and transitions. We have a distribution of absolute values of the splitting between the two modes $\left(|\Omega_{1}-\Omega_{2}|\right)$ with $\Omega_{Z}^{\|}=0.43\Omega_{ex}^{a}$ in $k_{z}=\pi /2a_{0}$ and $k_{z}=0$. Furthermore, we obtain that $E_{N}\left(\rho \right)$ tends to zero at T→0, as expected in the range where quantum fluctuations in T=0 are large. The behavior at range of higher *T* is only qualitative due to limitations in the spin wave approach used. In general, the behavior of quantum correlations is determined by the behavior of the energy bands that depend on coupling parameters and that generate a large effect on quantum entanglement.

### 3.4. Magnon Nernst Effect

The magnon Hall effect refers to the generation of magnon currents transverse to the applied electric field. The magnons act in response to the external field, impacting the magnon Hall conductivity, where the topological properties of magnon bands ν=α,β can be described by the Berry curvature $B^{\nu }\left(k\right)$, which can give rise to anomalous Hall-like responses even in the absence of net magnetization. $B^{\alpha (\beta )}\left(k\right)$ is defined as

(10) (Bν(k))ij=∑μ≠ν(σ3)νμ2Im[(jiks)νμ(σ3)μμ(∇jHk)μν][(σ3)ννΩν(k)−(σ3)μμΩμ(k)]2,

where $j_{jk}^{s}=\frac{1}{4}\left(\nabla_{j}H_{k}\sigma_{3}S^{s}+S^{s}\sigma_{3}\nabla_{j}H_{k}\right)$ is the polarized spin current and $\sigma_{3}=\mathrm{diag}\left(1,-1\right)⊗I_{N\times N}$ denotes the bosonic commutator, where the bosonic Hamiltonian can be written as $H=X^{†}HX$, where the basis X obeys the commutator relation $[X,X^{†}]=\gamma$, where $\gamma =Q\left[Y,Y^{†}\right]Q^{†}=Q\sigma_{3}Q^{†}$ and $Y=Q^{-1}X$ since *Q* is a paraunitary matrix and $\left[Y,Y^{†}\right]=\sigma_{3}$.

The spin Nernst coefficient $\alpha_{N}^{s}$ is provided by [25,26,27]

(11) $$\begin{array}{c} \alpha_{N}^{s}=\frac{2k_{B}T}{V}\sum_{\nu =\alpha ,\beta }\sum_{k}c_{1}\left(p\left(\Omega_{\nu k}\right)\right)B^{\nu }\left(k\right), \end{array}$$

where *V* is the volume of the system and $c_{1}\left(x\right)=\left(1+x\right)\ln\left(1+x\right)-x\ln x$, being the integral of the Berry curvature that is performed in the first Brillouin zone.

**Figure 3:**
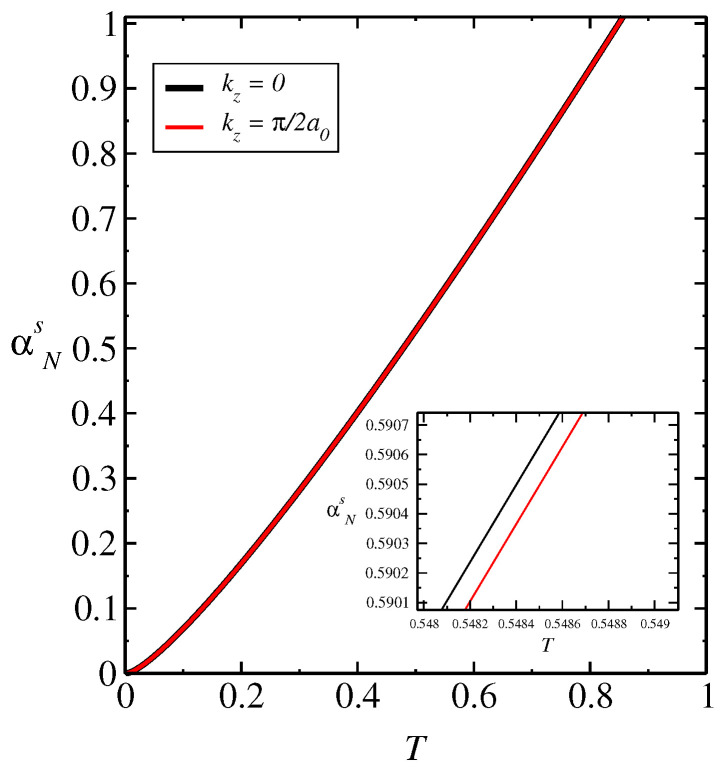
$\alpha_{N}^{s}$ as a function of *T* for $k_{z}=\pi /2a_{0}$, $k_{z}=0$, and $\Omega^{\left\|(⊥\right)}=0.43\Omega_{ex}^{a}$. We have a dependence on $J_{0}$ anisotropy constant $K_{a(d)}$ of $\Omega_{ex}^{a}$ provided by $J_{0}=\Omega_{ex}^{a}/2Z_{ad}S_{d(a)}$$K_{a(d)}=0.45\Omega_{ex}^{a}/S_{a(d)}$.

**Figure 4:**
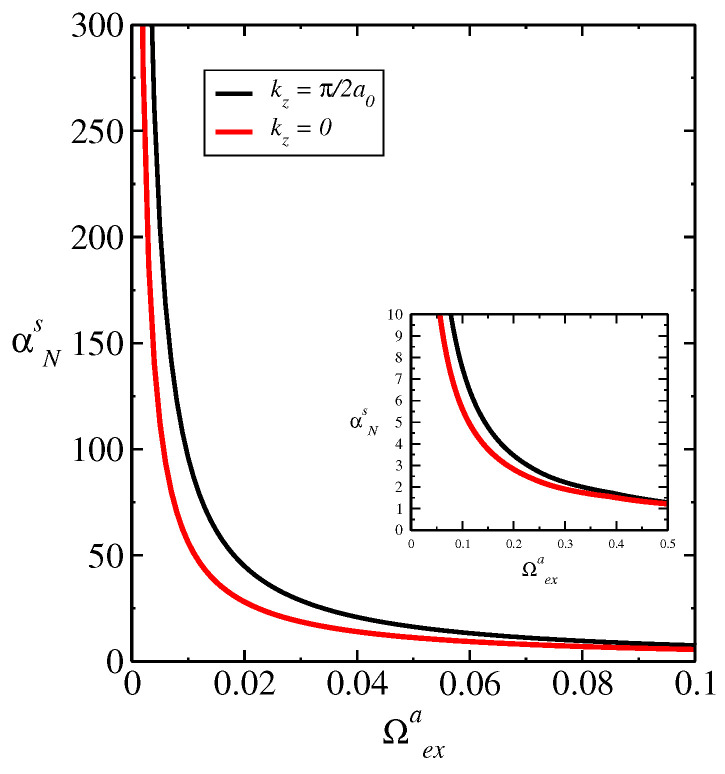
$\alpha_{N}^{s}$ as a function of $\Omega_{ex}^{a}$ for T=0.1J held fixed for $k_{z}=\pi /2a_{0}$, $k_{z}=0$, and $\Omega^{\left\|(⊥\right)}=0.43\Omega_{ex}^{a}$. We have a dependence of $\Omega_{ex}^{a}$ on $J_{0}$ and anisotropy constant $K_{a(d)}$ provided by $J_{0}=\Omega_{ex}^{a}/2Z_{ad}S_{d(a)}$$K_{a(d)}=0.45\Omega_{ex}^{a}/S_{a(d)}$.

In Figure 3, we obtain $\alpha_{N}^{s}$ as a function of *T* for $k_{z}=0$ and $k_{z}=\pi /2a_{0}$, which corresponds to the Brillouin zone edge in the *z* direction. We have $\Omega^{\left\|(⊥\right)}=0.43\Omega_{ex}^{a}$. We obtain that the very small variation in the curves of the spin Nernst coefficient $\alpha_{N}^{s}$ as a function of *T* for different values $k_{z}=0$ and $k_{z}=\pi /2a_{0}$ displayed in Figure 3, in the gap closing loop and where the system suffers a topological phase transition, generates a similar influence on the behavior of the curves of entanglement negativity $E_{N}$ vs. *T*. In general, the spin Nernst effect introduces non-equilibrium spin dynamics in the system, affecting the quantum correlations between spins, where, depending on the strength of the spin–orbit coupling and the temperature gradient, the spin Nernst effect may influence the entanglement generation, propagation, or decay in the ferrimagnetic material. Thus, the interplay of spin Nernst effect, anisotropy, and quantum correlations may give rise to novel phenomena such as spin Hall effect described by the spin Nernst coefficient. The very small variation in the behavior of $\alpha_{N}^{s}$ with $k_{z}$ is shown in the inset of Figure 3, indicating a small effect of the different magnon bands induced by $k_{z}$ on $\alpha_{N}^{s}$. In Figure 4, we obtain $\alpha_{N}^{s}$ as a function of $\Omega_{ex}^{a}$ for T=0.1J, $k_{z}=\pi /2a_{0}$, $k_{z}=0$, and $\Omega^{\left\|(⊥\right)}=0.43\Omega_{ex}^{a}$. Since $\Omega_{ex}^{a}$ depends on $J_{0}$ and biquadratic term $K_{a(b)}$, $J_{0}=\Omega_{ex}^{a}/2Z_{ab}S_{b(a)}$, $K_{a(b)}=0.45\Omega_{an}^{a}/S_{a(b)}$, we obtain a dependence of $\alpha_{N}^{s}$ with $J_{0}$ and $K_{a(b)}$ in the same way. We obtain a small variation in the curves for different $k_{z}$, as shown in the inset of the figure. The small variation obtained in the negativity and spin Nernst coefficient is a consequence of the small variation in the spin wave spectra with different intensities of external field along the easy axis indicated by $\Omega_{Z}^{\|}$.

## 4. Outlook

In brief, we analyzed the influence of dipole–dipole interactions on the spin Nernst coefficient and quantum entanglement in a lattice model provided by the layered ferrimagnet. Materials such as $Mn_{4}N$ and GdFe are some potentially relevant ferrimagnetic materials that exhibit skyrmions or PT-symmetric behavior of the emerging research. The quantities reported as well as the contribution to the Nernst coefficient of a given plane in *k*-space and entanglement negativity are experimentally relevant [2,58]. It is worth noting that the specific details and predictions regarding quantum correlations and the spin Nernst effect would depend on the specific model, Hamiltonian, and parameters used in the study. We established that the changes reported in the quantities analyzed are very small, where the contributions from the analyzed planes in *k*-space are relevant or dominant [5]. Moreover, the small changes in the curves of the spin Nernst coefficient $\alpha_{N}^{s}$ as a function of *T* for different values of $k_{z}=0$ and for $k_{z}=\pi /2a_{0}$, where the system suffers a topological phase transition, generate the same influence regarding the behavior of the curves of the entanglement negativity, $E_{N}$ vs. *T*. In a general way, the interplay of the spin Nernst effect, entanglement negativity, and magnetism is a complex topic that requires further investigation and research. It is plausible that the spin Nernst effect influences the entanglement properties of the layered ferrimagnet by affecting the spin dynamics and correlations within the system. However, the specific details and quantitative aspects of this influence depend on the particular characteristics of the material as well as its lattice structure and magnetic interactions.

## Figures and Tables

**Figure A1:**
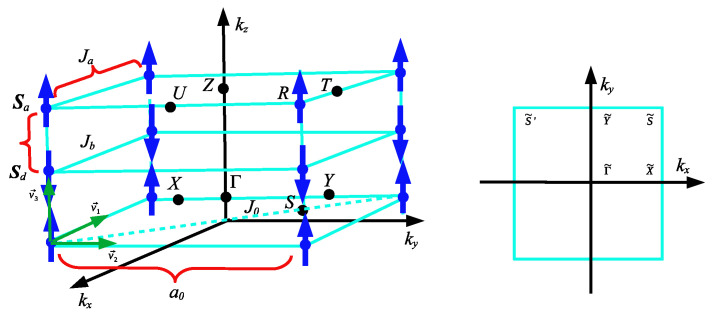
Lattice model for a layered ferrimagnet with ferromagnetic layers along *z* axis. $J_{0}$ is the interlayer coupling; $J_{a}$ and $J_{d}$ indicate the exchange interactions between nearest neighbors. The red arrows are the basis vectors, defined as ${\vec{v}}_{1}=\left(a_{0},0,0\right)$, ${\vec{v}}_{2}=\left(0,a_{0},0\right)$, and ${\vec{v}}_{3}=\left(0,0,2a_{0}\right)$, with $a_{0}$ being the in-plane lattice spacing.

## Data Availability

The data presented in this study are available on request from the corresponding author.

## Appendix A

*Layered ferrimagnet with single-ion anisotropy K, external field with dipole–dipole interaction*: The model is provided by the Hamiltonian

(A1) $$\begin{array}{c} \;H=\sum_{\langle \ell ,i,\ell ,j\rangle }J_{\ell }S_{\ell ,i}\cdot S_{\ell ,j}+J_{0}\sum_{\langle \ell ,i,\ell^{\prime },i\rangle }S_{\ell ,i}\cdot S_{\ell^{\prime },i}\\ +\sqrt{\frac{2k}{\mu_{0}}}\sum_{\ell ,i}S_{\ell ,i}\cdot B-\sum_{\ell ,i}K_{\ell }{\left(S_{\ell ,i}\cdot \hat{k}\right)}^{2}\\ +k\sum_{\left(\ell ,i\right)\neq \left(\ell^{\prime }j\right)}\frac{S_{\ell ,i}\cdot S_{\ell^{\prime },j}-3\left(S_{\ell ,i}\cdot {\hat{r}}_{ij}^{\ell \ell^{\prime }}\right)\left(S_{\ell^{\prime },j}\cdot {\hat{r}}_{ij}^{\ell \ell^{\prime }}\right)}{|r_{ij}^{\ell \ell^{\prime }}{|}^{3}}, \end{array}$$

where the unit vector $\hat{k}$ is in the direction of the easy axis, specified to *z* (*x*) for the in-plane (out-of-plane) magnetized geometry, and $r=|r_{ij}|$ is the distance between them. The representation of the lattice considered is provided in Figure A1. $K_{\ell }$ is the single-ion anisotropy and $J_{\ell }=J_{a}\left(J_{d}\right)$ are the ferrimagnetic intralayer exchanges. The notation used *ℓ* and *i* in $S_{\ell ,i}$ to denote the layer and the site, respectively, being $K_{\ell }=K_{a}\left(K_{d}\right)$ and $J_{\ell }=J_{a}\left(J_{d}\right)$ for the sublattice a (d). Moreover, $J_{0}$, $J_{a}$, and $J_{d}$ indicate the exchange constants between nearest neighbors. The unit vector $\hat{n}$ is the direction of the easy axis, which is specified to *x*(z) direction in-plane (out-of-plane).

*HP transformation:* We performed the Holstein–Primakoff (HP) transformation expanded up to first order after performing a local rotation of the spin operators of the model above to the new operators ${\tilde{S}}_{aj}^{+}$, ${\tilde{S}}_{aj}^{-}$, and ${\tilde{S}}_{ja}^{z}$[5] so that the mean-field direction of the spins points along the local *z* axis as follows:

(A2) $$\begin{array}{c} {\tilde{S}}_{aj}^{+}\approx \sqrt{2S}a_{j},\;{\tilde{S}}_{aj}^{-}\approx \sqrt{2S}a_{j}^{†},\;{\tilde{S}}_{ja}^{z}=S_{a}-a_{j}^{†}a_{j}, \end{array}$$

where we obtain the magnon Hamiltonian in the form of creation and annihilation operators $a_{j}^{†}\left(b_{j}^{†}\right)$ and $a_{j}\left(b_{j}\right)$, respectively.

*Semimetal phase in collinear configuration:* Whether the magnetic field is applied along the easy axis with intensity below the spin flop transition, we have a perpendicularly magnetized geometry φ=0. The ferrimagnetic state is in the collinear configuration with the spins aligning in the *z* direction, corresponding to $\varphi_{a}=\varphi_{d}=\pi /2$. The addition of the dipole–dipole interaction opens an anti-crossing gap at the intersection regions of the $k_{z}$ plane within the Brillouin zone, while the bands display linear crossings at the Brillouin zone edge, reflected in the role of dipole–dipole interaction as a source of magnon spin–orbit coupling [5,61] and nontrivial magnon band topology [62,63]. We consider the dipole–dipole interaction as a perturbation, considering its smaller magnitude compared to the other energy terms and projecting the magnon Hamiltonian into a reduced subspace with the basis consisting of the eigenstates of the Hamiltonian excluding the dipole–dipole interaction.

In the collinear configuration, we take the discrete Fourier transform of the boson operators and the Bogoliubov transformation to obtain the dispersion relation of magnons [5]

(A3) $$\begin{array}{c} \omega_{\alpha (\beta )}\left(k\right)=\frac{1}{2\hbar }\sqrt{{\left(a\left(k\right)\right)}^{2}+{\left(b\left(k\right)\right)}^{2}+2a\left(k\right)b\left(k\right)-4{\left(c\left(k\right)\right)}^{2}}\\ \pm \frac{1}{2\hbar }\left[a(k)-b(k)\right], \end{array}$$

where

(A4) $$\begin{array}{c} a\left(k\right)/\hbar =-\Omega_{ex}^{a}\cos\left(\varphi_{a}+\varphi_{b}\right)-\Omega_{ex}^{aa}\left[1-g^{aa}\left(k\right)\right]\\ +\frac{1}{2}\Omega_{an}^{a}\left(3\sin^{2}\varphi_{a}-1\right)-\left(\Omega_{Z}^{\|}\sin\varphi_{a}+\Omega_{Z}^{⊥}\cos\varphi_{a}\right),\\ b\left(k\right)/\hbar =-\Omega_{ex}^{d}\cos\left(\varphi_{a}+\varphi_{d}\right)-\Omega_{ex}^{dd}\left[1-g^{dd}\left(k\right)\right]\\ +\frac{1}{2}\Omega_{an}^{d}\left(3\sin^{2}\varphi_{d}-1\right)+\left(\Omega_{Z}^{\|}\sin\varphi_{d}-\Omega_{Z}^{⊥}\cos\varphi_{d}\right),\\ c\left(k\right)/\hbar =\frac{\Omega_{ex}^{ad}}{2}\left[1-\cos\left(\varphi_{a}+\varphi_{b}\right)\right]g^{ad}\left(k\right), \end{array}$$

and $\Omega_{ex}^{a(d)}=2J_{0}Z_{ad}S_{d(a)}$, $\Omega_{ex}^{ab}=2J_{0}Z_{ad}\sqrt{S_{d}S_{a}}$, $\Omega_{ex}^{aa(dd)}=2J_{a(d)}Z_{aa(dd)}S_{a(d)}$, $\Omega_{an}^{a(d)}=2K_{a(d)}S_{a(d)}$. Furthermore, we have $\Omega_{Z}^{\left\|(⊥\right)}=g\mu_{B}B_{\left\|(⊥\right)}$. $B_{\|}\left(B_{⊥}\right)$ corresponding to external field component along the easy axis. In addition, we have the form factors

$$\begin{array}{c} g^{aa}\left(k\right)=g^{dd}\left(k\right)=\frac{1}{2}\left[\cos\left(k_{x}a_{0}\right)+\cos\left(k_{y}a_{0}\right)\right],\\ g^{ad}\left(k\right)=\cos\left(k_{z}a_{0}\right),\;k_{z}=\frac{\pi }{2a_{0}}, \end{array}$$

where $a_{0}$ is the lattice spacing constant.
